# A Psycho-Socio-Cultural Investigation of Factors Involved in the *Perezhivanie* of Chilean Mothers

**DOI:** 10.11621/pir.2025.0208

**Published:** 2025-06-01

**Authors:** Juan J. Rubio-González, Catalina Escobar Torres, Valentina Flores Avalos, Daniela Jofré Godoy

**Affiliations:** a University of Atacama, Copiapó, Chile

**Keywords:** *perezhivanie*, motherhood, psychological factors, social factors, cultural factors

## Abstract

**Background:**

Motherhood in Chile is complex and challenging - marked as it is by a growing number of women raising children on their own, against a backdrop of multiple social, cultural and economic factors- as well as a significant decline in birth rates. Perezhivanie is a Russian word, originating in Russian scholarship. This concept integrates the personal, social, and environmental dimensions of individual experience. This study adopts a cultural-historical approach grounded in Vygotskian theory, proposing perezhivanie as the unit of analysis.

**Objective:**

To study the *perezhivanie* of Chilean women in relation to the psychological, social, and cultural factors underlying the complexities of motherhood.

**Design:**

This study uses a qualitative epistemology and adopts a constructive-interpretative paradigm proposed by González-Rey. As a research strategy, it uses a case study design and applies a purposive sampling method which allows to recruit 10 Chilean mothers to whom the episodic interview technique proposed by Flick is applied. Data analysis was conducted following González-Rey’s method to interpreting subjective productions.

**Results:**

The main findings indicate that participants construct a perezhivanie marked by guilt and frustration within a social framework characterised by family difficulties, of monoparenting, and lack of economic resources, all associated with cultural patterns that reinforce the unequal distribution of parenting responsibilities and gender stereotypes in society.

**Conclusion:**

The study concludes on the importance of studying and providing comprehensive responses to the complex reality of motherhood in Chile.

## Introduction

According to figures from the Chilean National Institute of Statistics (NIS, 2022), the average age of women having their first child in Chile increased by 2.57 years between 1972 and 2016. This same institute confirms the downward trend in births in Chile, a phenomenon that has been occurring since the mid-20th century. Additionally, the proportion of monoparent family households stands at 27.4%, with 82% of them headed by a woman raising her children alone (NIS, 2020). In this context, Mendoza & Saldivia (2015) point out that most of the problems in motherhood are related to the mother’s socioeconomic means.

The antecedents described configure a complex scenario for Chilean women to take decisions about motherhood. In this sense, it must be considered that in this country the prevalence of depression during pregnancy affects one in five mothers, while anxious symptoms have a 41.3% prevalence during the perinatal period (Coo et al., 2021). Along these same lines, scientific literature shows that the problem in motherhood is also related to other psychosocial and cultural factors. The most notable of these are the personality characteristics of the mother, the socioeconomic and cultural context in which pregnancy occurs, and the woman’s planning and management of her pregnancy and subsequent motherhood as regards psychic phenomena (Cáceres et al., 2014). In addition, research on postpartum depression in Chile suggests that while the incidence of new depressive symptoms is consistent across socioeconomic levels, the prevalence is higher among women from lower-income backgrounds. This is likely due to pre-existing mental health conditions rather than socioeconomic status being a direct cause of postpartum depression (Cisternas, 2006).

In addition to the above, there are complex processes that occur during gestation. Most notable include the management of the emotional impact of perinatal losses, sociodemographic characteristics and the existence or otherwise of social support perceived by the pregnant woman (Damato, 2000; Doan & Zimerman, 2003). Mothers must go through unpleasant emotional states, caused by unstable relationships, low educational and socioeconomic levels, compounded by the rejection from their social environment regarding motherhood in certain circumstances, especially when the pregnancy is neither desired nor supported by their social network (Alhusen et al., 2012). In that regard, motherhood in Chile is embodied by women who some studies describe as fighters and who, in the process of pregnancy and upbringing, develop various psychosocial problems (Pérez-Díaz & Oyarce, 2020). The most notable of these are burnout and the phenomenon of mental load due to gender roles that women must comply with (Fuentes et al., 2010), difficulties in the mother-child bond and alterations in care and parenting patterns (Cáceres et al., 2014; Massey et al., 2015; Pisoni et al., 2014).

In relation to the social support perceived by mothers, informal support stands out, made up of primary networks, such as the couple, family, friends, and community in general (Colangelo, 2020), as well as formal social supports consisting of state institutions involved in maternal protection (Álvarez & Poblete, 2023). In these social supports, stressors such as domestic violence to which some pregnant women are exposed play a fundamental role (Mayor & Salazar, 2019). This phenomenon is sometimes associated with a desexualisation of women, who are stripped of desire and eroticism in this phase, coupled with an environment that tends not to validate their emotions (Cieza, 2019). Additionally, the situation of poverty and exclusion experienced by some mothers varies from living on the streets, having to work and care for their children alone (Genolet et al., 2009; Piqueras et al., 2020; Tacca et al., 2020), to those who must deal with the upbringing of children with physical or mental health difficulties (Ferreira & Najar, 2018).

In addition, there are the cultural factors involved in motherhood, framed in a patriarchal structure (Lerner, 1990), which establishes marked gender roles associated with upbringing, where women assume a submissive, service-oriented role and are receptive to family responsibilities (Márquez et al., 2021). In this context, machismo stands out, reinforcing aversion towards women, being a factor that legitimises violence against them (Ferrer & Bosch, 2000), which can be observed in another cultural pattern such as the sexual contract, which deepens asymmetry in the intimacy of couples, establishing exclusive ideals for the male sex regarding sexual fulfillment and satisfaction (Lagarde, 1997; Pateman, 1995). In addition, the discrimination that mothers experience in the labour and institutional spheres is evident, as they tend to be perceived as threats to labour organisations on one hand (Riquelme, 2011), a matter that translates into constant harassment by their superiors (NIS, 2022). In institutional terms, this discrimination is exemplified by symbolic obstetric violence, which De Bruyn (2003) defines as a set of abusive practices that physically and psychologically violate women due to their motherhood condition and can trigger discomfort during the gestation, childbirth, and postpartum periods.

This detailed reality contrasts with the requirements of international organisations, such as the proposal at the United Nations Millennium Summit, which calls on its member countries to improve maternal health and reduce infant mortality during the early decades of the 21st century (González et al., 2013). Based on the reviewed literature, various issues associated with motherhood in Chile are evident. These involve psychological, economic, political, and cultural factors that shape women’s subjectivity as they recount their experiences (Palomar, 2005). In the light of this, the present study adopts *perezhivanie* as its unit of analysis, a concept which, according to Vygotsky (1994), reflects the relationship between the individual and their environment. That is, *perezhivanie* captures how a person experiences, interprets, and emotionally engages with a given event or lived experience, thus enabling a comprehensive processing of such experiences (Blunden, 2016; Rubio et al., 2023). Within this framework, the study aims to examine the *perezhivanie* of Chilean women in relation to the psychological, social, and cultural factors underlying the challenges of motherhood.

## Theoretical background

From the cultural-historical perspective rooted in Vygotskian theory, it is established that activities carried out by human beings are historically situated, culturally mediated, and socially executed (Rodríguez, 2013). This implies addressing social processes in a contextualised manner and establishing continuities when analysing the experiences lived by individuals, who evolve and develop as historical and social beings, as well as being active agents in the construction of their subjectivity (Vygotsky, 1994).

In this regard, Vygotsky (1996) states that the term of *perezhivanie* (*perezhivaniya* in plural) is the true dynamic unity of consciousness, a unity that constitutes the basis of consciousness…” (p. 383). This is based on the logic that people play an active role and establish a dialectical relationship with their environment; just as they are capable of transforming their environment, it also transforms them. In particular, Vygotsky (1994) states that the psychological configurations that individuals develop are related to how they experience their lives at particular moments of developmental process. These formulations originate from Vygotsky’s (1994) clinical studies with children experiencing various physical and psychological difficulties, through which he established a particular relationship between individuals and their environment. Within this context, the category of *perezhivanie* illustrates how individuals assign personal meaning to their experiences, thereby defining a specific and unique connection between themselves and their context.

Specifically, Vygotsky (1996) noted that it is difficult to determine whether *perezhivanie* stems from the influence of the environment or constitutes a quality of that environment. What he affirms, however, is that *perezhivanie* represents the unity between personality and environment: “There is no *perezhivanie* without motive, just as there is no conscious act that is not an act of consciousness of something. Nevertheless, every *perezhivanie* is personal” (Vygotsky, 1996, p. 383). In this sense, *perezhivanie* refers to how a person becomes aware of, interprets, and emotionally engages with a particular event (Vygotsky, 1994a, p. 341). Thus, *perezhivanie* embodies a complex unit of analysis that encompasses internal and external factors, as well as the sociocultural environment at a specific moment in an individual’s life (Bozhovich, 2009; Fleer et al., 2017).

## Methods

The research was positioned within a qualitative epistemology and the constructive-interpretative paradigm, as proposed by Cuban psychologist Fernando González-Rey (1997; 2009). This methodological perspective aims to address complex phenomena related to human subjectivity (Macedo et al., 2016) and has been primarily developed in psychology research oriented towards the cultural-historical approach. In this sense, qualitative epistemology and the constructive-interpretative paradigm are inspired by the Vygotskian logic of highlighting the dialectical relationship between sociocultural contexts, emotional, and cognitive processes of individuals, emphasising the social and systemic nature of the human mind (Díaz & González-Rey, 2005; González-Rey, 1997; 2009). Particularity is valued as a fundamental source of knowledge, not so much for its individuality, but for the meaning it contributes to the construction of theoretical models (González-Rey, 1997; 2009). Thus, the constructive and interpretative nature of human knowledge is emphasised, conceived as an active process of production rather than a simple passive assimilation of reality by individuals (González-Rey, 1997; 2009).

The research process is conceived as a dynamic dialogue where the researcher interprets and formulates conclusions from the collected data, thus generating theoretical models related to the study topic, which allows for opening new horizons for research, knowledge, and professional practices (Goulart et al., 2019; Macedo et al., 2016). In terms of design, the research adopts a case study approach (Yin, 2009), as a methodological strategy, that allows for a particular, complex, systematic, and in-depth exploration of the phenomenon under study.

## Participants, data collection, and analysis

To select the participants, a case sampling method was used with previously defined criteria (Flick, 2019). This strategy made it possible to select 10 female participants, between 20 and 76 years old, from the towns of Copiapó, Caldera and Tierra Amarilla, in the Atacama region of Chile. They included students, workers and homeowners who had experienced difficulties in their motherhood. The participants were invited to the interview, and they were informed regarding the purpose of the study, and the procedure of data collection. They also were asked to sign written consent forms prior to their participation in this study. Subsequently, multiple meetings were held with each participant, until the information was collected. It is important to note that the use of information complied with Chilean Law 19.628 (1999), which protects the personal data of participants and establishes that their participation must be based on prior, informed, and written consent.

For data collection, episodic interviewing was used, which, following Flick’s proposal (2019), allowed access to the narrative, emotional, episodic, semantic, and symbolic content of the participants regarding their complex experiences with motherhood. In this regard, the interviewers were trained in the procedure and the technique, with each interview lasting an average of two hours per participant. It is worth noting that one of the strengths of the episodic interview is its capacity to enable triangulation within the same interview, as participants produce both semantic-symbolic and episodic-emotional knowledge within a single narrative act (Flick, 2019).

According to the epistemology and investigative paradigm, the collected data were analysed using the proposal of interpretation of subjective productions (Díaz & González-Rey, 2005) because the data obtained in this study represented a process of subjectivisation, in which the participants constructed multiple meanings, so they were later worked on for their reconstruction and interpretation (Goulart et al., 2019). It was necessary for both the audio recordings and the texts of the interviews to be reviewed several times to understand the emotional tone and the content of the reflections of the participants. This repetition in the analysis allowed for evaluating the reflective and discursive constructions, giving them theoretical meaning, as it is in this act where the participants’ discursive intention is configured (Díaz & González-Rey, 2005). In this process, themes or patterns were developed that represented the *perezhivaniya* of the participants and served as the basis for the construction of the theoretical model that emerged from this analysis.

## Results

The *perezhivanie* of their emotional and psychological experiences about motherhood reflects a complex mix of guilt, frustration, excitement, and happiness, especially when motherhood was a personal choice. These emotions are shaped by a desire to break generational patterns and see motherhood as both a challenge and a chance for personal growth. In this context, physiological changes during and after childbirth contributed to emotional distress, particularly when paired with issues like anxiety, disordered eating, and low self-esteem. Pre-existing mental health struggles, such as affective blocks, self-harm, and suicidal ideation, often worsened during motherhood, especially when participants felt societal pressure to take on the role of mother.

For some participants, the trauma of past violence and sexual abuse, especially during adolescence, was magnified by pregnancy, marking motherhood as a process characterised by psychological pain. Health issues in their children, such as neurodivergence, further contributed to emotional strain, though some participants also reported the development of coping resources like patience and self-regulation. In that sense, the maternal-foetal bond was shaped by the context of pregnancy. Unwanted pregnancies often led to ambivalent or delayed bonding, while pregnancies following abuse sometimes strengthened the bond as a protective mechanism. Traumatic birth experiences were also common, often associated with fear, loneliness, and a sense of loss rather than celebration, due to the sense of disillusionment with the idealised image of the process in contrast to its reality.

Another element that impacts the emotional stability of these women is the unequal division of domestic responsibilities, which further aggravates the mental burdens. Participants felt overextended, as their partners often viewed helping with household duties as a favour, rather than a shared responsibility. In this context, the generation of mother-child bonds, as well as the cognitions and emotions, are marked by subjective constructions where gender roles, intrafamilial and institutional violence, prejudices and discriminations, monoparenting, and inequitable mental burden, among other factors, are highly relevant

Socially, participants experiences were shaped by family conflict, single parenting, financial stress, and weak support systems. Some participants reported judgment or hostility from family members, especially in cases of unplanned pregnancy, leading to emotionally strained environments that limited their capacity to parent. In this sense, the absence or abandonment of partners especially during critical moments like a child’s illness intensified feelings of stress, disillusionment, and anxiety. In some cases, older daughters took on parental roles, resulting in parentified children and altered family dynamics.

Cycles of domestic violence, emotional, physical and sexual abuse were common, often reflecting some of the participants’ own childhood experiences. Breaking these cycles was described as lonely but liberating. However, societal stigma against single mothers added a layer of judgment and isolation. In that context, financial concerns were a major source of stress, particularly for women raising children alone or caring for neurodivergent children, which often required expensive treatments. Survivors of violence frequently had to work multiple jobs to support their families.

Participants also felt misunderstood and unsupported by society, especially when navigating the diagnosis of a child or resisting pressure to maintain harmful relationships. Some had experienced forced marriages, often following teenage pregnancies or abuse, underscoring the moral and cultural pressures placed on them. In addition to the above, institutional mistreatment particularly in healthcare and education further added to their sense of vulnerability. Many reported dehumanising treatment during childbirth and felt blamed for their children’s mental health challenges. However, some found solidarity and practical support in their local communities, particularly among women, which offered emotional strength and a sense of safety.

It can be seen that as a result of the cultural and social burden, the socio-affective aspects that revolve around families are centered on the figure of the mother. This, as a result of these conditions, builds her identity and advances in her life in a contradictory manner: she generates resources to the extent that she has social support, but stagnates when she does not have them.

Culturally, the *perezhivanie* of the participants was shaped by gender norms and expectations around motherhood. Many felt burdened by having to shoulder most parenting responsibilities alone, driven by societal views that frame caregiving and domestic tasks as exclusively female roles. They also felt pressured to meet idealised standards of motherhood without having been taught how to navigate that role. Cultural scripts that prescribe a linear path of marriage, pregnancy, then motherhood were experienced as constraining, and breaking from these norms often resulted in criticism or rejection.

Participants also report that they had to balance motherhood with work and that their relationships generated anxiety and identity loss, as their personal identities became eclipsed by the maternal role. Some were even forced into marriage with their abusers to preserve family reputation, leading to profound emotional trauma. In this regard, some participants also reported misogyny and sexual coercion within their relationships, particularly when asserting autonomy. Acts of violence and jealousy were often normalised as expressions of love.

Discrimination in the workplace due to motherhood was another recurring theme, including exclusion during hiring processes and denial of maternity rights. In some cases, even other women contributed to this discrimination, creating feelings of betrayal and disappointment. As a result, many participants expressed hesitance or regret around motherhood, citing its negative impact on their personal and professional lives. In this logic, the lived experiences of these women demonstrate the marked relevance of social support, as well as certain aspects of the patriarchal culture, which are entrenched in the micro and macro social and institutional spaces and operate as devices that hinder the normal development of the mother role and permeate the construction of these identities.

## Discussion

Following the logic of the analysis of subjective interpretations proposed by Díaz & González-Rey (2005), after evaluating the reflexive and discursive constructions of the participants and giving them theoretical sense, it is possible to formulate that motherhood is a process that develops in constant feedback with context, but at the same time, complex and contradictory. This is due to the permanent tension between the expectations of these mothers and the demands of the environment, added to the cultural and transgenerational patterns with which they live and must deal with during pregnancy and subsequent child-rearing.

In relation to the aforementioned, Doan & Zimerman (2003) highlight the importance of self-care in motherhood, a factor that when it was not present in the case of this study, the participants realised that they had self-harming behaviours. Likewise, mental workload emerges as a determinant component in the psychological well-being of mothers, corroborating the findings of Fuentes et al. (2010) on the unequal distribution of cognitive tasks associated with the organisation of the household. Although the factors are consistent with cultural patterns, there are others of an institutional nature such as discrimination in health, education and workplaces, which operate as social stressors as pointed out by De Bruyn (2003).

Regarding the social factors involved in motherhood, the results reflect the validity of Alvarez & Poblete’s (2023) approach on the relevance of social support and stressors in emotional stability. Mothers reported a range of social difficulties that significantly complicate the exercise of motherhood. On this point, the study also validates what De Bryun (2003) pointed out, regarding the violence of institutionalised discrimination against women in different contexts such as the workplace. In addition to this, the experiences of domestic violence are determining factors that intensify the vulnerability of mothers, reaffirming what Mayor & Salazar (2019) proposed about its character as a direct social stressor.

Regarding the cultural factors involved in motherhood, the participants reported a strong influence of traditional gender roles, with women being almost exclusively assigned domestic and caregiving tasks. This confirms the findings of Márquez et al. (2021) regarding the persistence of cultural patterns that naturalise this unequal distribution. At the same time, expressions of machismo and misogyny were identified in various dimensions, including sex-affective life, a situation that reflects social hostility towards women who seek independence and autonomy, as argued by Ferrer & Bosch (2003).

## Conclusion

Given the objective of understanding the *perezhivanie* of Chilean women with respect to the psychological, social and cultural factors linked to the challenges of motherhood, the findings of this research offer a comprehensive response to the complex reality of being a mother in Chile. The simultaneous attention to the multiple factors they face in their daily lives allows for a broader, evidence-based view to understand human behaviour in its dialectical connection with society and culture. Likewise, the *perezhivanie* unit of analysis makes it possible to interpret these women’s subjective constructions of motherhood and the situational and contextual factors that shape it. The results suggest the design and implementation of state programmes aimed at promoting maternal mental health, with a humanised prenatal care approach, reflective and sensitive to women’s experiences, thus reducing the suffering associated with motherhood, especially in contexts of vulnerability.

## Limitations

Since this is a qualitative study, the sample is limited and confined to a specific region. Future research, from a quantitative approach, could expand the contexts and sample size in order to collect systematic data and contrast research hypotheses. It would also be pertinent to focus attention on legislation, regulations and institutional protocols that impose and reinforce sociocultural patterns linked to motherhood. In this way, future research could influence public policies and legislative frameworks at the national level. It is also crucial to address the psychological, social and cultural consequences for children who have lived through complex experiences with their mothers. Finally, it is important to delve deeper into the personal and collective resources that mothers develop. One of them aims to investigate cooperative or “tribal” child-rearing practices, which embody a communitarian logic opposed to the individualistic models predominant in today’s society.
